# Persistence on Therapy and Propensity Matched Outcome Comparison of Two Subcutaneous Interferon Beta 1a Dosages for Multiple Sclerosis

**DOI:** 10.1371/journal.pone.0063480

**Published:** 2013-05-21

**Authors:** Tomas Kalincik, Timothy Spelman, Maria Trojano, Pierre Duquette, Guillermo Izquierdo, Pierre Grammond, Alessandra Lugaresi, Raymond Hupperts, Edgardo Cristiano, Vincent Van Pesch, Francois Grand’Maison, Daniele La Spitaleri, Maria Edite Rio, Sholmo Flechter, Celia Oreja-Guevara, Giorgio Giuliani, Aldo Savino, Maria Pia Amato, Thor Petersen, Ricardo Fernandez-Bolanos, Roberto Bergamaschi, Gerardo Iuliano, Cavit Boz, Jeannette Lechner-Scott, Norma Deri, Orla Gray, Freek Verheul, Marcela Fiol, Michael Barnett, Erik van Munster, Vetere Santiago, Fraser Moore, Mark Slee, Maria Laura Saladino, Raed Alroughani, Cameron Shaw, Krisztian Kasa, Tatjana Petkovska-Boskova, Leontien den Braber-Moerland, Joab Chapman, Eli Skromne, Joseph Herbert, Dieter Poehlau, Merrilee Needham, Elizabeth Alejandra Bacile Bacile, Walter Oleschko Arruda, Mark Paine, Bhim Singhal, Steve Vucic, Jose Antonio Cabrera-Gomez, Helmut Butzkueven, on behalf of the MSBase Study Group, Elaine Roger, Pierre Despault, Mark Marriott, Anneke Van der Walt, John King, Trevor Kilpatrick, Katherine Buzzard, Vilija Jokubaitis, Jill Byron, Lisa Morgan, Olga Skibina, Jodi Haartsen, Giovanna De Luca, Valeria Di Tommaso, Daniela Travaglini, Erika Pietrolongo, Maria di Ioia, Deborah Farina, Luca Mancinelli, Damiano Paolicelli, Pietro Iaffaldano, Juan Ignacio Rojas, Liliana Patrucco, Etienne Roullet, Jorge Correale, Celica Ysrraelit, Cartechini Elisabetta, Eugenio Pucci, David Williams, Lisa Dark, Vahid Shaygannejad, Cees Zwanikken, Norbert Vella, Carmen-Adella Sirbu

**Affiliations:** the Centre hospitalier del’Universite de Montreal, Hopital Notre-Dame, Canada; the Centre hospitalier del’Universite de Montreal, Hopital Notre-Dame, Canada; the Royal Melbourne Hospital, Australia; the Royal Melbourne Hospital, Australia; the Royal Melbourne Hospital, Australia; the Royal Melbourne Hospital, Australia; the Royal Melbourne Hospital, Australia; the Royal Melbourne Hospital, Australia; the Royal Melbourne Hospital, Australia; the Royal Melbourne Hospital, Australia; Box Hill Hospital, Monash University, Australia; Box Hill Hospital, Monash University, Australia; Department of Neuroscience and Imaging, University ‘G. d’Annunzio’, Italy; Department of Neuroscience and Imaging, University ‘G. d’Annunzio’, Italy; Department of Neuroscience and Imaging, University ‘G. d’Annunzio’, Italy; Department of Neuroscience and Imaging, University ‘G. d’Annunzio’, Italy; Department of Neuroscience and Imaging, University ‘G. d’Annunzio’, Italy; Department of Neuroscience and Imaging, University ‘G. d’Annunzio’, Italy; Department of Neuroscience and Imaging, University ‘G. d’Annunzio’, Italy; University of Bari, Italy; University of Bari, Italy; Hospital Italiano, Argentina; Hospital Italiano, Argentina; Hopital Tenon, Paris, France; FLENI, Argentina; FLENI, Argentina; Ospedale di Macerata, Italy; Ospedale di Macerata, Italy; John Hunter Hospital, Australia; John Hunter Hospital, Australia; Al-Zahra Hospital, Iran; MS-Centrum Nijmegen, Nijmegen, The Netherlands; Mater Dei Hospital, Malta; Central Clinical Emergency Military Hospital; 1 Departments of Medicine and Neurology, University of Melbourne and Royal Melbourne Hospital, Melbourne, Australia; 2 Department of Neurology, Royal Melbourne Hospital, Melbourne, Australia; 3 Department of Basic Medical Sciences, Neuroscience and Sense Organs, University of Bari, Bari, Italy; 4 Hôpital Notre Dame, Montreal, Canada; 5 Hospital Universitario Virgen Macarena, Sevilla, Spain; 6 Hotel-Dieu de Levis, Quebec, Canada; 7 MS Center, Department of Neuroscience and Imaging, University ‘G. d’Annunzio’, Chieti, Italy; 8 Maaslandziekenhuis, Sittard, The Netherlands; 9 Hospital Italiano, Buenos Aires, Argentina; 10 Cliniques Universitaires Saint-Luc, Brussels, Belgium; 11 Neuro Rive-Sud, Hôpital Charles LeMoyne, Quebec, Canada; 12 AORN San Giuseppe Moscati, Avellino, Italy; 13 Hospital S. Joao, Porto, Portugal; 14 Assaf Harofeh Medical Center, Beer-Yaakov, Israel; 15 University Hospital San Carlos, IdISSC, Madrid, Spain; 16 Ospedale di Macerata, Macerata, Italy; 17 Consultorio Privado, Buenos Aires, Argentina; 18 Department NEUROFARBA, Section of Neurology, University of Florence, Florence, Italy; 19 Kommunehospitalet, Aarhus C, Denmark; 20 Hospital Universitario Virgen de Valme, Seville, Spain; 21 Neurological Institute IRCCS Mondino, Pavia, Italy; 22 Ospedali Riuniti di Salerno, Salerno, Italy; 23 Karadeniz Technical University, Trabzon, Turkey; 24 John Hunter Hospital, Newcastle, Australia; 25 Hospital Fernandez, Buenos Aires, Argentina; 26 Craigavon Area Hospital, Portadown, United Kingdom; 27 Groen Hart Ziekenhuis, Gouda, The Netherlands; 28 FLENI, Buenos Aires, Argentina; 29 Brain and Mind Research Institute, Sydney, Australia; 30 Jeroen Bosch Ziekenhuis, ‘s-Hertogenbosch, The Netherlands; 31 HIGA Gral, San Martin La Plata, Argentina; 32 Jewish General Hospital, McGill University, Montreal, Canada; 33 Flinders University and Medical Centre, Adelaide, Australia; 34 INEBA, Buenos Aires, Argentina; 35 Amiri Hospital, Kuwait City, Kuwait; 36 Geelong Hospital, Geelong, Australia; 37 Jahn Ferenc Teaching Hospital, Budapest, Hungary; 38 Clinic of Neurology Clinical Center, Skopje, Macedonia; 39 Francicus Ziekenhuis, Roosendaal, The Netherlands; 40 Sheba Medical Center, Tel Hashomer, Israel; 41 Hospital Angeles Mexico City, Lomas, Mexico; 42 New York University Hospital for Joint Diseases, New York, New York, United States of America; 43 Multiple Sclerosis Centre Kamillus-Klinik, Asbach, Germany; 44 Royal North Shore Hospital, Sydney, Australia; 45 Instituto de Neurociencias Cordoba, Cordoba, Argentina; 46 Hospital Ecoville, Curitiba, Brazil; 47 St Vincent’s Hospital, Melbourne, Australia; 48 Bombay Hospital Institute of Medical Sciences, Mumbai, India; 49 Westmead Hospital, Sydney, Australia; 50 Centro Internacional de Restauracion Neurologica, Havana, Cuba; 51 Department of Neurology, Box Hill Hospital and Monash University, Melbourne, Australia; University Hospital Basel, Switzerland

## Abstract

**Objectives:**

To compare treatment persistence between two dosages of interferon β-1a in a large observational multiple sclerosis registry and assess disease outcomes of first line MS treatment at these dosages using propensity scoring to adjust for baseline imbalance in disease characteristics.

**Methods:**

Treatment discontinuations were evaluated in all patients within the MSBase registry who commenced interferon β-1a SC thrice weekly (n = 4678). Furthermore, we assessed 2-year clinical outcomes in 1220 patients treated with interferon β-1a in either dosage (22 µg or 44 µg) as their first disease modifying agent, matched on propensity score calculated from pre-treatment demographic and clinical variables. A subgroup analysis was performed on 456 matched patients who also had baseline MRI variables recorded.

**Results:**

Overall, 4054 treatment discontinuations were recorded in 3059 patients. The patients receiving the lower interferon dosage were more likely to discontinue treatment than those with the higher dosage (25% vs. 20% annual probability of discontinuation, respectively). This was seen in discontinuations with reasons recorded as “lack of efficacy” (3.3% vs. 1.7%), “scheduled stop” (2.2% vs. 1.3%) or without the reason recorded (16.7% vs. 13.3% annual discontinuation rate, 22 µg vs. 44 µg dosage, respectively). Propensity score was determined by treating centre and disability (score without MRI parameters) or centre, sex and number of contrast-enhancing lesions (score including MRI parameters). No differences in clinical outcomes at two years (relapse rate, time relapse-free and disability) were observed between the matched patients treated with either of the interferon dosages.

**Conclusions:**

Treatment discontinuations were more common in interferon β-1a 22 µg SC thrice weekly. However, 2-year clinical outcomes did not differ between patients receiving the different dosages, thus replicating in a registry dataset derived from “real-world” database the results of the pivotal randomised trial. Propensity score matching effectively minimised baseline covariate imbalance between two directly compared sub-populations from a large observational registry.

## Introduction

Primary evidence of therapeutic efficacy is provided by randomised controlled trials (RCT). However, RCTs require substantial amount of resources, are time-consuming, associated with significant costs and employ highly specific selection criteria. Therefore, patients included in RCTs might not be representative of the general MS population. Additionally, many potential treatment comparisons will never be subjected to RCTs because of lack of commercial interest and large sample sizes required to show a difference.

Multicentre observational databases have the potential to describe large, longitudinally evaluated and prospectively assessed cohorts representative of general populations with specific conditions. The MSBase registry is an international, observational database collecting longitudinal data from a large population of patients with multiple sclerosis (MS; n = 18,886 in February 2012). This patient population is representative of patients managed in academic MS centres, which typically also recruit patients for RCTs. [1] Analyses of treatment outcomes in observational registries such as MSBase are susceptible to significant biases, e.g. confounding by treatment indication, recall bias or detection bias. [2] In such analyses, appropriate methods of bias reduction are required and need to be validated. The propensity scoring method is commonly employed to estimate the effect of multiple potential confounders on treatment assignment. [3], [4] The result, a single propensity score per case, is then used to adjust for individual confounders of treatment assignment through subject selection, matching or outcome weighting [5]–[7].

The pivotal RCT of interferon (IFN) β-1a SC three times weekly vs. placebo (Prevention of Relapses and Disability by IFN β-1a Subcutaneously in MS, PRISMS) provided the primary evidence of its clinical effect in relapsing-remitting MS. [8] In this RCT, clinical efficacy was no different between the two tested dosages (22 µg vs. 44 µg). After documenting treatment persistence of first-line use of these IFN dosages in the MSBase dataset, we assessed clinical outcomes between two propensity score-matched subpopulations of patients treated with either of the dosages as first line therapy and compared these results to those obtained in the PRISMS RCT.

## Patients and Methods

### Ethics Statement

The MSBase registry was approved by the Melbourne Health Human Research Ethics Committee, and by the local ethics committees in all participating centres (or exemptions granted, according to applicable local laws and regulations). If required, written informed consent was obtained from enrolled patients.

### Database and Study Population

Data extracted from MSBase in February 2012 comprised longitudinal clinical data of more than 100,000 patient-years from 18,886 patients from 55 MS centres in 25 countries. All subjects with data recorded within the MSBase registry who received at least one dose of IFNβ-1a SC (Rebif; Merck Serono, Geneva, Switzerland) prior to February 2012 were included in the treatment discontinuation analysis.

The primary analysis of treatment outcomes was performed in patients treated with first-line Rebif in either available dose (i.e. 22 µg or 44 µg SC three times weekly) for at least two consecutive years, with no previous exposure to other disease modifying or immunosuppressive therapy and without switching between the doses. A prerequisite was availability of demographic and clinical information (including measures of disability and relapse activity) throughout the two-year follow-up period. Patients were excluded on the basis of long disease duration (>10 years from disease onset) and low disease activity (no relapses within the two years preceding baseline), in order to approximate the PRISMS study population.

A secondary analysis was performed in a subset of patients with investigator-classified cerebral MRI scans within the two years prior to the baseline visit. This subset was used to calculate a different propensity score including the MRI variables.

### Data Acquisition

The data were recorded in a prospective, observational manner, as a part of routine clinical practice. Information about MS-related outcomes was updated during clinic visits, using the iMed patient record system to enter data at each of the participating centres. Disability was scored by accredited scorers using the Expanded Disability Status Scale (EDSS). Quality of the EDSS assessment was assured by the requirement of online Neurostatus certification at each of the participating centres. Date of onset of clinical relapses was recorded. Annualised relapse rate (ARR) was calculated based on the relapse onsets recorded within the year preceding treatment initiation (baseline relapse activity) and the two years following the baseline (on-treatment relapse activity). Duration of MS was estimated as the time since the patient-reported first clinical manifestation of the disease (recorded retrospectively). The presence, relationships and number of relatives with the diagnosis of MS was recorded in a proportion of patients. MRI brain scans were performed as part of routine clinical practice at each of the participating centres. Availability of T2-weighted imaging with locally reported number of hyperintense cerebral T2 lesions (categorised as 1–8 or 9+ per scan) was the minimum prerequisite for inclusion in the secondary analysis. If gadolinium-containing contrast was administered according to local procedures, gadolinium-enhancing lesions (Gd+) were evaluated as present or absent.

To assure quality of the analysed data, only information from centres with at least 10 active records was used, as stipulated in the study protocol. The minimum prerequisite was at least annual data updates. For all events, including new symptoms, clinical relapses, quantification of disability, changes in disease course, MRI and laboratory investigations and adverse events, a date of event onset was required. Prior to analysis the recorded data were verified using a series of automated procedures to identify any invalid or inconsistent entries.

### Analysis of Treatment Discontinuation and Switch

Statistical analyses were carried out using Statistica 10 (Statsoft, Tulsa, OK, USA) and R software (http://www.R-project.org). Incidence of treatment discontinuation events with respect to the recorded reasons for discontinuation was compared between the treatment dosages using the Andersen-Gill models with Efron approximation method. These models are used to model time to recurrent events, compensating for highly variable treatment exposure and the fact that each subject could consecutively receive multiple treatments. The models were adjusted for patient age, sex and country. In selected variables, a “missing” value was allowed in order to avoid patient exclusions. Cases were censored at the time of the last visit unless the time of treatment discontinuation event was specified. Goodness of model fit was evaluated using the Akaike information criterion. Initiation of Rebif 44 µg within a month of discontinuing Rebif 22 µg was considered as treatment escalation. Similarly, treatment with Rebif 22 µg within a month of discontinuing Rebif 44 µg was viewed as treatment de-escalation.

### Analysis of Treatment Outcomes

Treatment outcomes were analysed within selected populations of patients (see above) matched based on their propensity of assignment to treatment dosage. All matching procedures were performed using R, the MatchIt package. [9] The propensity score was calculated using a logistic regression model with the outcome variable represented by assignment to the Rebif dosage (with Rebif 22 µg set as the reference category). The model excluding MRI data was built using the following variables: age, disease duration, ARR, EDSS category, disease course, number of relatives with MS and MS centre. The model including the baseline MRI data contained two additional variables, the number of cerebral T2 lesions (categorical, 1–8 or 9+) and the Gd+ lesion status (not given, 0 or 1+). No interaction terms were included. The individual propensity scores (with and without MRI findings) were calculated as weighted sums of those variables with non-zero weights (at 0.1 level of statistical significance).

Patients in the two treatment groups were then matched in a 1∶1 ratio using nearest neighbour matching without replacement and discarding from both groups the cases outside the common support of the distance measure (i.e. the common hull of the pooled propensity scores). [10], [11] Closeness of the match between the matched patients was evaluated using cumulative and average distances, analysis of standardised differences and tests of statistical significance (paired t-test and McNemar test). After assessing normality of data distribution, treatment outcomes were compared between the propensity score-matched patients with Wilcoxon signed-rank test (EDSS, change in EDSS and ARR) and McNemar test (relapse status) as appropriate. Time free from relapse was estimated by Kaplan-Meier analysis and proportions of relapse-free patients were compared between the groups with Log-rank test censored at two years. Cumulative hazard of multiple relapses was estimated and compared between the groups with the Andersen-Gill model (see above). Since the differences in the baseline variables were accounted for during the matching procedure, no further adjustments for potential confounders were performed. All reported p-values are two-tailed and for each analysis p≤0.05 was considered significant. The number of hypothesis-testing procedures was low, therefore no adjustment for multiple hypothesis testing was applied. Power within the used statistical models was estimated.

## Results

### Discontinuation of Treatment

**Figure 1:**
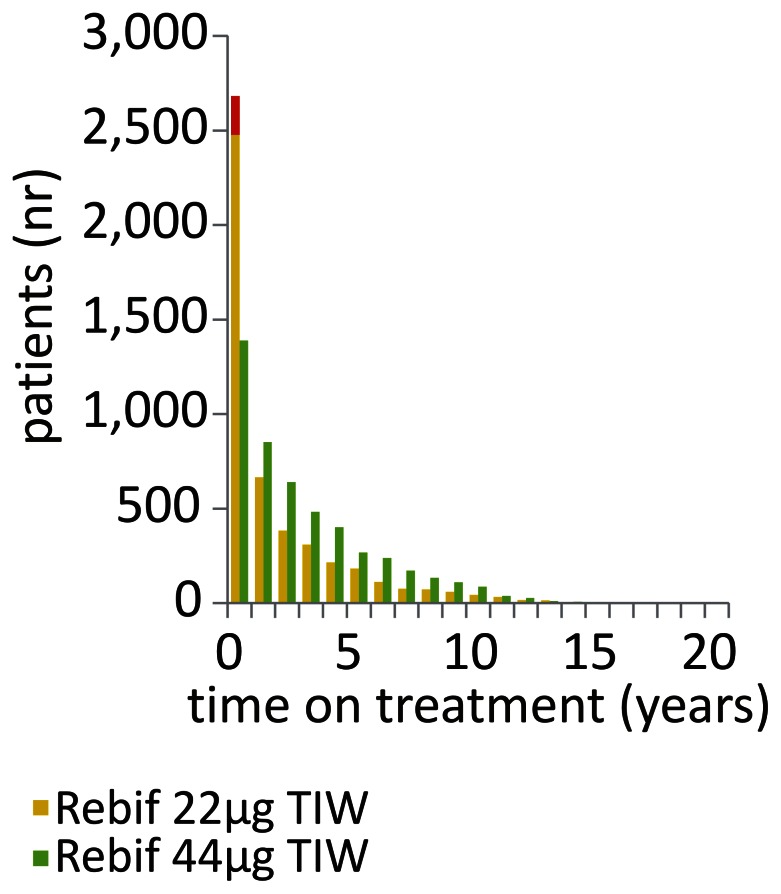
Exposure to treatment with interferon β-1a SC thrice weekly. Numbers of patients treated with Rebif recorded within the MSBase registry (n = 4678) and stratified by time on treatment are shown. Red bar in year 1 indicates the proportion of patients in whom dose escalation was a planned procedure. TIW, three times weekly.

**Figure 2:**
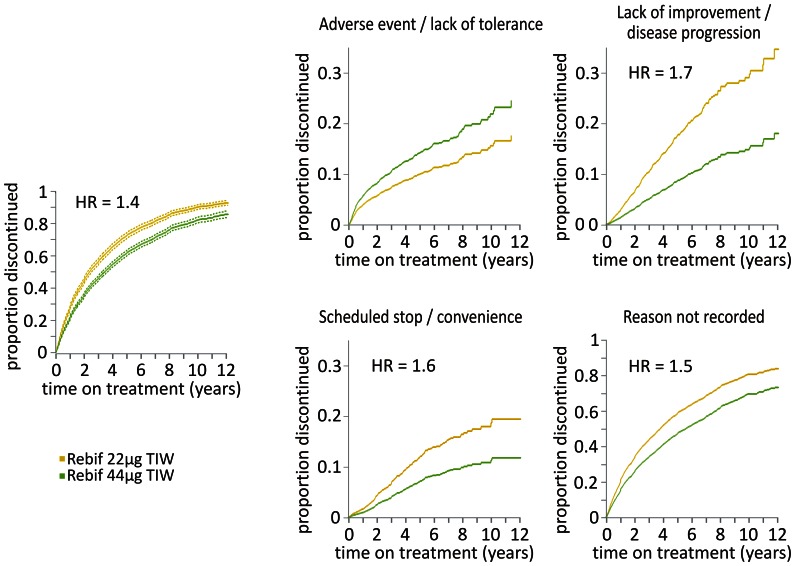
Likelihood of discontinuation by exposure to treatment. Overall proportion of treatment discontinuations in patients treated with either Rebif dosage is shown (left). Discontinuation rates by the recorded reasons are shown. Hazard ratio (HR) is given where significantly different from 1, dashed lines represent 95% confidence intervals. Planned dose escalations within the first year of treatment are not included. HR, hazard ratio; TIW, three times weekly.

Among the 18,886 patients included in the MSBase registry as of February 2012, we identified 4678 patients exposed to Rebif. Of these, 1188 (72% females) were treated with the 22 µg dosage, 2488 (71% females) were treated with the 44 µg dosage and 1002 (72% females) patients received both the dosages at various times. The average patient age was 36±10 years and disease duration was 7±7 years (mean ± SD), for both treatment dosages at the time of their first initiation. Median treatment period was 2.1 and 2.5 years for the 22 µg and 44 µg dosages, respectively. Total patient years of follow up were 6480 for the 22 µg and 11,432 for the 44 µg dosage. Distribution of time on treatment is shown in Figure 1. It can be seen that the number of patients treated with the 22 µg dose for less than 1 year was disproportionately high compared to the longer treatment durations. In total, 4054 treatment discontinuations were recorded in 3059 patients, 1808 from Rebif 22 µg and 2246 from Rebif 44 µg. There were 192 dosage escalations occurring within the initial 12 months of treatment with Rebif 22 µg, and these were excluded from further analyses (red bar in Figure 1). Table 1 provides an overview of the recorded reasons for treatment discontinuation. It is worth noting that in a substantial proportion of cases, the reason for discontinuation was not specified (68%). The annual probability of treatment discontinuation reached 25% in patients on Rebif 22 µg and 20% in patients on Rebif 44 µg. For more detailed list of annual probabilities categorised by the recorded reasons for discontinuation, see Table 1. After adjusting for time on treatment, age, sex and country, the patients treated with Rebif 22 µg were more likely to discontinue treatment than those with Rebif 44 µg (hazard ratio (HR) = 1.4, p = 10^−16^, Andersen-Gill model, see Figure 2). This difference was apparent in the sub-group analysis with the reason for discontinuation specified as lack of improvement/progression of disease (HR = 1.7, p = 10^−6^), scheduled stop/convenience (HR = 1.6, p = 0.001) or without the reason recorded (HR = 1.5, p = 10^−16^). In contrast, the discontinuation rates due to adverse events/lack of tolerance did not significantly differ between the treatment groups (p = 0.98, Andersen-Gill models).

**Table 1:** Discontinuation events.

	all patients	Rebif 22 µg	Rebif 44 µg
adverse event	313 (1.7%)	111 (1.7%)	202 (1.8%)
lack of tolerance	243 (1.4%)	61 (0.9%)	182 (1.6%)
lack of improvement	205 (1.1%)	109 (1.7%)	96 (0.8%)
progression of disease	208 (1.2%)	108 (1.7%)	100 (0.9%)
scheduled stop	117 (0.7%)	55 (0.8%)	62 (0.5%)
convenience	177 (1.0%)	88 (1.4%)	89 (0.8%)
N/A	2599 (14.5%)	1084 (16.7%)	1515 (13.5%)
**Total**	**3862 (21.6%)**	**1616 (24.9%)**	**2246 (19.9%)**

Data are presented as number of discontinuation events with annual probability of discontinuation stratified by recorded reasons for discontinuation. The events were recorded in all patients within the MSBase ever treated with Rebif. Escalations of treatment dosage planned as part of the treatment initiation protocol (i.e. occurring within the initial 6 months of treatment with Rebif 22 µg) were excluded.

Of the recorded discontinuation events, 466 were evaluated as escalations of Rebif dosage (including the 192 escalations occurring within the initial year of treatment). Apart from the 356 events with the reason not recorded, the most frequent reason for escalation was lack of improvement/progression of disease (94). Similarly, 123 discontinuation events were considered to be de-escalations of the Rebif dosage. The reason was not specified in 79 cases and an adverse event/lack of tolerance was recorded in 41 cases.

### Disease Outcomes: Validation of Propensity Matched Outcome Analysis

#### Primary analysis

To directly compare clinical outcomes of treatment with Rebif 22 µg and 44 µg as the first disease modifying treatment used for at least two consecutive years, 614 and 682 patients were selected, respectively (for baseline characteristics see Table 2). The propensity score (i.e. the likelihood of assignment to the 44 µg Rebif dosage) not including any MRI parameters was determined predominantly by the MS centre (OR = 0.05–15, p≥10^−7^, logistic regression, see Table S1). In addition, the score was increased by the absence of neurological disability (i.e. by EDSS step 0; OR = 1.8, p = 0.07). After applying the nearest matching procedure, 610 patients were retained in each of the treatment groups. Summative distance between the propensity scores of the matched groups decreased from 229 to 159, with the average pairwise distance decreasing from 0.34±0.12 to 0.26±0.13 per patient (mean ± SD). Characteristics of the matched patients are given in Table 2. No marked differences in the recorded variables were seen between the matched groups.

**Table 2:** Baseline demographic and clinical data in patients unmatched and matched by the propensity score.

		Unmatched	Matched				
		Rebif 22 µg	Rebif 44 µg	Rebif 22 µg	Rebif 44 µg	Cohen d	p
subjects (females)		614 (69%)	682 (68%)	610 (68%)	610 (69%)		NS
age (mean ± SD)		34.7±9.7	35.5±9.7	34.7±9.6	35.7±9.7	0.11	NS
MS duration (mean ± SD)		4.0±2.5	4.1±2.6	4.0±2.5	4.2±2.6	0.08	NS
annualised relapse rate (mean ± SD)		1.3±1.0	1.3±1.0	1.3±1.0	1.4±1.0	0.02	NS
EDSS (median (interquartile range))		2 (1.5, 3.5)	2 (1.5, 3.5)	2 (1.5, 3.5)	2 (1.5, 3.5)		
EDSS category	[0]	4%	9%	4%	7%		NS
	[1–1.5]	23%	29%	23%	28%		
	[2–2.5]	37%	29%	38%	30%		
	[3–3.5]	20%	15%	20%	15%		
	[4–9.5]	15%	17%	15%	19%		
MS course	[CIS]	5%	5%	5%	5%		NS
	[RRMS]	92%	91%	92%	90%		
	[SPMS]	3%	3%	3%	3%		
	[PPMS]	1%	1%	1%	1%		
number of relatives with MS	[0]	92%	94%	92%	94%		NS
	[1]	7%	4%	7%	5%		
	[2+]	1%	2%	1%	1%		

CIS, clinical isolated syndrome; EDSS, Expanded Disability Status Scale; MS, multiple sclerosis; PPMS, primary progressive multiple sclerosis; RRMS, relapsing-remitting multiple sclerosis; SD, standard deviation; SPMS, secondary progressive multiple sclerosis.

**Figure 3:**
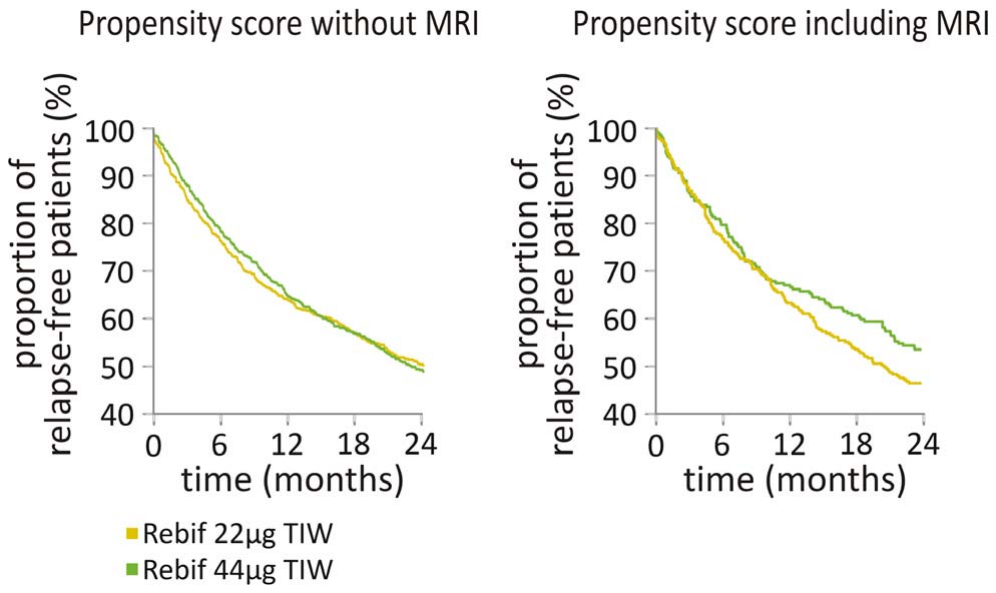
Kaplan-Meier plots for the proportion of patients free from clinical relapses. No statistically significant differences between the treatment dosages were observed. MRI, magnetic resonance imaging; TIW, three times weekly.

Table 3 compares the clinical outcomes between the matched groups after two years of treatment with either Rebif dosage. Neither EDSS nor ARR differed significantly between the groups (p≥0.5, signed-rank test). ARR was reduced by 66% and 68% compared to baseline in the lower and the higher dosage groups, respectively. Proportions of patients free from relapses after two years were 49% and 50% in the Rebif 22 µg and 44 µg groups, respectively (p = 0.8, McNemar test), with time to first relapse (p = 0.9, Log-rank test, see Figure 3) and cumulative risk of relapses comparable between the treatment groups (p = 0.5, Andersen-Gill model). Power contained within the statistical models was sufficient to uncover treatment effects of the following sizes at 90% power and the specified level of statistical significance: EDSS, 0.25; change in EDSS, 0.18; ARR, 0.09; cumulative relapse risk, 0.1.

**Table 3:** Clinical outcomes at two years of treatment in the patient groups matched on propensity scores.

	All matched patients (n = 1220)		Matched subgroup with MRI data (n = 452)	
	Rebif 22 µg	Rebif 44 µg	Rebif 22 µg	Rebif 44 µg
**annualised relapse rate**				
mean±SD	0.4±0.6	0.4±0.6	0.4±0.5	0.4±0.6
median (interquartile range)	0.5 (0, 0.5)	0 (0, 0.5)	0.5 (0, 0.5)	0 (0, 0.5)
relative ARR reduction	66%	68%	72%	71%
**time to first relapse**				
mean±SD [months]	16.5±9.0	16.2±9.3	16.0±8.9	16.6±9.1
proportion relapse-free	49%	50%	46%	51%
**EDSS**				
mean±SD	2.6±1.6	2.5±1.7	2.4±1.6	2.3±1.7
median (interquartile range)	2 (1.5, 3.5)	2 (1.5, 3.5)	2 (1.5, 3)	2 (1, 3.5)
**EDSS change**				
mean±SD	0.1±1.2	0.1±1.2	0.0±1.3	0.1±1.2
median (interquartile range)	0 (−0.5, 1)	0 (−0.5, 0.5)	0 (−0.5, 0.5)	0 (−0.5, 1)

All matched patients and the subset with available MRI data are shown.

ARR, annualised relapse rate; EDSS, Expanded Disability Status Scale; MRI, magnetic resonance imaging; SD, standard deviation.

#### Secondary analysis

The propensity score involving semi-quantitative MRI parameters at baseline was determined predominantly by the MS centre (OR = 0.2–7, p≥0.0001, logistic regression). In addition, men (OR = 2, p = 0.002) and patients with 9 or more T2 lesions (OR = 1.8, p = 0.09) were more likely to receive Rebif 44 µg. The matching procedure retained 226 patients in each group, with summative distance between the propensity scores of the groups decreasing from 105 to 44 and the average pairwise distance decreasing from 0.36±0.12 to 0.2±0.1 per patient (mean ± SD). Table 4 provides group characteristics before and after matching. Despite the overall decrease in distance between the two dosage groups, statistically significant differences in age and the number of hyperintense T2-lesions were not eliminated by the matching procedure.

**Table 4:** Baseline demographic, clinical and MRI data in the sub-group with available MRI, unmatched and matched by the propensity score.

		Unmatched	Matched				
		Rebif 22 µg	Rebif 44 µg	Rebif 22 µg	Rebif 44 µg	Cohen d	p
subjects (females)		269 (75%)	294 (65%)	226 (70%)	226 (67%)		NS
age (mean ± SD)		33.8±8.9	35.1±10	33.2±8.9	35.1±9.9	0.20	0.03
MS duration (mean ± SD)		4.0±2.5	3.9±2.4	4.1±2.5	4.0±2.4	0.05	NS
annualised relapse rate (mean ± SD)		1.5±1.0	1.4±1.0	1.5±1.0	1.4±1.0	0.06	NS
EDSS (median (interquartile range))		2.5 (1.5, 3)	2 (1, 3)	2 (1.5, 3)	2 (1, 3.5)		
EDSS category	[0]	5%	11%	6%	8%		NS
	[1–1.5]	24%	29%	28%	29%		
	[2–2.5]	31%	35%	36%	34%		
	[3–3.5]	15%	25%	19%	17%		
	[4–9.5]	14%	11%	12%	12%		
MS course	[CIS]	4%	7%	5%	7%		NS
	[RRMS]	94%	91%	93%	90%		
	[SPMS]	1%	2%	2%	2%		
	[PPMS]	0%	0%	0%	0%		
number of relatives with MS	[0]	92%	93%	91%	93%		NS
	[1]	7%	5%	8%	6%		
	[2+]	1%	0%	1%	0%		
MRI brain: T2 lesions	[1]–[8]	93%	70%	91%	77%		0.02
	[9+]	7%	30%	9%	23%		
MRI brain: Gd+ lesions	[0]	88%	73%	87%	80%		NS
	[1+]	10%	21%	12%	16%		
	[missing]	1%	6%	1%	4%		

CIS, clinical isolated syndrome; EDSS, Expanded Disability Status Scale; MRI, magnetic resonance imaging; MS, multiple sclerosis; PPMS, primary progressive multiple sclerosis; RRMS, relapsing-remitting multiple sclerosis; SD, standard deviation; SPMS, secondary progressive multiple sclerosis.

Clinical outcomes in this analysis inclusive of baseline MRI were similar to the outcomes of the larger comparative analysis detailed above (Table 3). Both EDSS and ARR at two years were comparable between the matched groups (p≥0.9, signed-rank test). ARR was reduced by 72% and 71% compared to baseline in the lower and the higher dosage groups, respectively. Proportions of patients free from relapses at two years were 46% and 51% in the Rebif 22 µg and 44 µg groups, respectively (p = 0.7, McNemar test), with time to first relapse (p = 0.1, Log-rank test, see Figure 3) and cumulative risk of relapses similar in both groups (p = 0.9; Andersen-Gill model). The models contained 90% power at the specified level of statistical significance to uncover effect sizes as follows: EDSS, 0.4; change in EDSS, 0.31; ARR, 0.13; cumulative relapse risk, 0.2.

## Discussion

Using data from a large clinical practice MS registry, MSBase, we have shown that patients with IFNβ-1a SC thrice weekly (Rebif) in the 22 µg dosage are more likely to discontinue treatment than those receiving Rebif in the 44 µg dosage. Annual discontinuation rates reached 25% and 20% in the two treatment dosages, respectively. Compared to Rebif 44 µg, the 22 µg dosage was more often discontinued due to perceived insufficient effect or a scheduled stop. In order to compare clinical outcomes of the original PRISMS trial with real-world practice, we performed propensity score-matched pairwise analyses of patients receiving either dosage of Rebif as first-line MS therapy who continued on their respective dosage for at least two years. In agreement with the PRISMS trial, our closely matched populations did not show any effect of Rebif dosage on two-year clinical outcomes.

The mean annual probability of discontinuing Rebif within the MSBase registry was 23%, which has markedly exceeded the treatment discontinuation rate reported in the PRISMS study (10–11% over two years). [8] Similarly, the annual discontinuation rates due to reported adverse events were marginally higher in our study compared to the PRISMS trial (3% and 1.5–2.4%, respectively). Interestingly, the PRISMS and the EVIDENCE trials reported a dose-dependent incidence of adverse events. Namely, decreases in leukocyte, neutrophil and lymphocyte counts, increase in aminotransferase levels and injection site reactions were found to be more frequent in the groups with higher dosages of IFNβ. [8], [12] In the present study, we have shown a similar trend towards higher annual discontinuation rates due to adverse events/lack of tolerance in patients receiving Rebif in the higher dosage, however, this did not reach statistical significance.

It could be argued that an expected better efficacy of the higher Rebif dosage (as perceived by patients and clinicians) could have inflated the discontinuation rate in the Rebif 22 µg group. In this case the discontinuation events would most likely be followed by dose escalations. Since the instances of increase in the Rebif dosage from 22 µg to 44 µg were not included in the analysis of discontinuation events, we assume that the effect of the perceived different therapeutic efficacy on treatment discontinuation was minimal. Overall, the dose escalation was a commonly observed phenomenon (466 cases, i.e. 26% of all discontinuation events in the Rebif 22 µg group). Even though lack of effect was the most commonly specified reason for escalation (in 20% of escalations), the reason was unspecified for 76% escalation events. It is worth noting that almost half of the escalations took place within the first year of treatment initiation, of which 83% were unspecified. We presume that a high proportion of the early escalations were likely planned as part of routine treatment initiation procedure used at some centres. In agreement with this is the observation that scheduled stop as a reason for discontinuation was more commonly recorded among patients treated with Rebif 22 µg.

Baseline characteristics of the MSBase cohort included in this study and the PRISMS study were remarkably similar. Patients had mean disease duration of 4 years in the MSBase study and 5.3 years in the PRISMS study, with the median EDSS of 2 and 2.5, respectively. Baseline mean ARR was only marginally different between the MSBase and PRISMS studies (1.3 vs. 1.5, respectively). Outcomes of the propensity-matched Rebif dosage comparison confirmed a lack of any statistically significant dose-dependent differences in relapse frequency or disability, as demonstrated in PRISMS. [8] Interestingly, our observed on-treatment ARR was 0.4 (for each dosage), while the PRISMS reported ARR of 0.91 and 0.86 after two years of treatment with Rebif 22 µg and 44 µg, respectively. If this difference is to be attributed to a potential under-reporting of relapses in the MSBase registry, it should be noted that this, if present, would in all likelihood apply to either of the treatment groups equally, and thus would be unlikely to confound the analysis comparing the outcomes of the two Rebif dosages. Reassuringly, our reported ARR is comparable to the ARR reported in patients receiving IFNβ-1a in the most recent RCTs (0.3–0.4). [13], [14] Also, the reduction of ARR (66–72%) and proportion of relapse-free patients (46–51%) at two years were substantially higher in our study than in the PRISMS trial (39–42% and 27–32%, respectively). Finally, we showed a much less pronounced increase in EDSS over two years (0–0.1) compared to the PRISMS study (0.23–0.24). The PRISMS trial also showed a dose-dependent effect of IFNβ-1a on MRI parameters, which we were not able to assess, as the quantitative MRI data are not routinely recorded in the MSBase registry. The major difference potentially accounting for these large absolute outcome differences between the MSBase study and the PRISMS randomised trial is the fact that we only included patients with a two-year treatment completion at either dose of Rebif. We know that annualised discontinuation rates of Rebif in the MSBase dataset amount to 23%, therefore the patients with poor relapse control were likely to be differentially lost from the two studies. Nonetheless, the results suggest high treatment efficacy over two years in real-world patients treated with Rebif (at either dose) as their first DMD.

Importantly, we were able to derive a large patient sub-population from the MSBase clinical practice registry with different initial treatment assignations (largely determined by centre preference) whose two-year outcomes could be compared using patient pairs that were determined with propensity-score baseline covariate matching. We obtained a similar primary result (i.e. the lack of dosage-dependent treatment effect) to that obtained in the pivotal randomised trial examining the same treatment outcomes. We therefore believe that imbalance within patient populations non-randomly assigned to different treatment can potentially be controlled with propensity-based methods. Such methods include weighting, stratification, matching and covariate adjustment. Studies in observational cohorts of patients with MS had previously employed propensity score-weighted analyses to evaluate disease outcomes, [5], [15]–[17] propensity score-based stratification to assess long-term benefits of early versus delayed immunomodulatory treatment [7], [18] and propensity score matching to evaluate sex difference in response to IFNβ. [19] Combinations of propensity score stratification with other methods, such as recursive partitioning, were also tested [6].

While our approach provided sufficient power for the subsequent analyses and resulted in a patient sample that was likely to be representative of patient populations at MS centres, it did not eliminate the bias potentially introduced by unknown confounders. To ameliorate this risk, we have accounted for the location-specific hidden confounders (e.g. centre-specific dose preferences) by adjusting our models for treating centre. As the matching algorithm, we have chosen the nearest neighbour procedure in a 1∶1 ratio with a relatively benevolent criterion for excluding the cases outside the hull of the pooled distance measure. [10] Even though this did not result in a perfect overlap of the propensity scores between the two matched populations, it still led to a marked decrease in the mean distance between the matched groups. For a perfect overlap to be achieved, a stricter matching criterion would have been required, which in turn would have resulted in exclusion of a high number of patients and unnecessary loss of power. We have therefore chosen to use the criterion that allowed us to preserve power while achieving a satisfactory match.

We also adjusted our statistical models for age, sex and country, which we have shown to be related to treatment discontinuation. [20] However, we were unable to adjust the analyses for change in disability, as this was usually not recorded at the time of treatment discontinuation. Moreover, we were unable to include information about relapse severity and recovery, which was often missing and the resulting statistical models would most probably be overfitted. A potential under-estimation of the frequency of treatment discontinuations due to specific reasons could stem from the relatively high proportion of discontinuation events with the reason not specified. Also, baseline cerebral MRI data were missing in the majority of patients. However, a propensity-matched subgroup analysis including MRI did not yield results different to the subgroup analysis excluding MRI. It is of note that the quality of the MRI data were likely to be variable, as they were provided by the clinicians using a semi-quantitative evaluation of MRI lesions carried out in a number of scanners with variable protocols. However, the number of hyperintense T2-lesions and the presence/absence of Gd+ lesions were probably the MRI characteristics that were most likely to influence clinical decision-making with respect to DMD choice. It should also be noted that the quality of clinical data recorded in observational registries such as MSBase is unlikely to be similar to the quality of data originating from RCTs during the on-treatment period. Paradoxically, the quality of data pertaining to the pre-treatment time is actually likely to be better, as it is generally prospectively recorded in MSBase prior to treatment start, whereas in clinical trials disease and relapse history is typically collected retrospectively. Finally, the inclusion criterion of sustained therapy with Rebif for at least two years resulted in bias towards selecting patients with more satisfactory treatment response. We presume that this bias influenced either of the dosage groups symmetrically and did not confound the comparison of disease outcomes between the groups.

## Conclusion

In this study, we have shown that direct real-world treatment comparisons can be conducted on registry data. Using the global MSBase registry data, we conducted a propensity score-based pairwise patient selection method to compare treatment outcomes between two doses of IFN β-1a thrice weekly (Rebif 22 µg vs. Rebif 44 µg). The dosage comparisons in our study with respect to differences in relapse rate and EDSS change mirrored those obtained from the pivotal RCT and enabled their broader generalisation. This method could be of increasing importance for head-to-head evaluation of the rapidly increasing number of disease modifying therapies in MS, many of which will never be compared to each other in RCTs. Although we do not claim that the results produced by the analyses of the observational registries can substitute for RCTs, we believe that the described technique represents a useful and feasible option when RCTs are not feasible or unlikely to be conducted.

## Supporting Information

Table S1 **Assignation to treatment dosage by treating centres.** The table shows number of patients assigned to either Rebif dosage at each of the participating centres. Odds relative to the reference centre (IT-002) of assignation to the higher dosage are given. The results were incorporated in the individual propensity scores.
(DOCX)
