# Supplementary material for: Persistence on Therapy and Propensity Matched Outcome Comparison of Two Subcutaneous Interferon Beta 1a Dosages for Multiple Sclerosis
Source: PLoS One. 2013 May 21;8(5):e63480. doi: 10.1371/journal.pone.0063480 (PMC3660604; doi:10.1371/journal.pone.0063480)
Supplement: Table S1 — Assignation to treatment dosage by treating centres. The table shows number of patients assigned to either Rebif dosage at each of the participating centres. Odds relative to the reference centre (IT-002) of assignation to the higher dosage are given. The results were incorporated in the individual propensity scores. (DOCX) [file pone.0063480.s001.docx]

**Supplementary table S1**

**Assignation to treatment dosage by treating centres**

| centre  code | Rebif 22 μg  (patients) | Rebif 44 μg  (patients) | Odds  ratio | Standard  error | p-value |
| --- | --- | --- | --- | --- | --- |
| AR-001 | 0 | 7 |  |  | 1.0 |
| AR-003 | 2 | 6 |  |  | 0.5 |
| AR-010 | 1 | 2 |  |  | 0.9 |
| AR-011 | 0 | 1 |  |  | 1.0 |
| AR-012 | 8 | 11 |  |  | 0.9 |
| AR-013 | 11 | 24 |  |  | 0.5 |
| AR-015 | 4 | 2 |  |  | 0.2 |
| AU-009 | 1 | 14 | 8.0 | 2.9 | 0.054 |
| AU-011 | 1 | 13 | 9.5 | 3.0 | 0.039 |
| AU-012 | 0 | 3 |  |  | 1.0 |
| AU-014 | 0 | 1 |  |  | 1.0 |
| AU-017 | 2 | 13 | 4.5 | 2.2 | 0.070 |
| AU-018 | 0 | 1 |  |  | 1.0 |
| AU-019 | 0 | 5 |  |  | 1.0 |
| BE-002 | 14 | 19 |  |  | 0.7 |
| CA-005 | 1 | 5 |  |  | 0.2 |
| CA-007 | 59 | 65 |  |  | 0.2 |
| CA-009 | 17 | 64 | 2.2 | 1.5 | 0.043 |
| CA-010 | 3 | 29 | 6.0 | 2.0 | 0.006 |
| CU-001 | 0 | 1 |  |  | 1.0 |
| DK-001 | 12 | 6 | 0.3 | 1.8 | 0.040 |
| ES-001 | 1 | 17 | 10.0 | 3.0 | 0.032 |
| ES-003 | 10 | 10 |  |  | 0.2 |
| ES-008 | 14 | 104 | 4.1 | 1.5 | 10^-4^ |
| FR-001 | 2 | 1 |  |  | 0.3 |
| HU-003 | 0 | 3 |  |  | 1.0 |
| IL-004 | 11 | 10 |  |  | 0.3 |
| IT-002 | 21 | 33 | * | * | * |
| IT-003 | 9 | 11 |  |  | 0.5 |
| IT-004 | 317 | 103 | 0.2 | 1.3 | 10^-7^ |
| IT-005 | 15 | 4 | 0.1 | 1.8 | 0.002 |
| IT-008 | 9 | 7 |  |  | 0.2 |
| IT-010 | 1 | 15 | 9.0 | 3.0 | 0.046 |
| IT-012 | 1 | 24 | 14.9 | 3.0 | 0.010 |
| MK-004 | 2 | 0 |  |  | 1.0 |
| NL-001 | 10 | 8 |  |  | 0.2 |
| NL-002 | 0 | 2 |  |  | 1.0 |
| NL-003 | 19 | 25 |  |  | 0.9 |
| NL-006 | 3 | 5 |  |  | 0.9 |
| PT-002 | 21 | 2 | 0.05 | 2.2 | 10^-4^ |
| TR-005 | 12 | 4 | 0.2 | 1.8 | 0.009 |
| US-002 | 0 | 2 |  |  | 1.0 |

* reference centre

The table shows number of patients assigned to either Rebif dosage at each of the participating centres. Odds relative to the reference centre (IT-002) of assignation to the higher dosage are given. The results were incorporated in the individual propensity scores.
